# Determinants of immunization status among 12–24 months old children in Ethiopia: Using 2019 Ethiopian mini demographic and health survey data

**DOI:** 10.1371/journal.pone.0283629

**Published:** 2023-03-30

**Authors:** Kassahun Animut Metkie, Getasew Berhanu Melese, Behailu Dessalegn W/silassie, Fatuma Ebrahim Ali

**Affiliations:** Department of Statistics, College of Natural and Computational Science, Dilla University, Dilla, Ethiopia; National Research Centre, EGYPT

## Abstract

**Background:**

Vaccination is a global success story, one of the most effective and successful health interventions for health and development, saving the lives of millions of children every year. In 2018, nearly 870,000 Ethiopian children did not receive the life-saving measles, diphtheria, and tetanus vaccines. This study aimed to determine what factors influence children’s immunization status in Ethiopia.

**Methods:**

Immunization status was examined in a sample of 1843 children aged 12–24 months using data from the 2019 Ethiopian Mini Demographic and Health Survey 2019. The study used percentages to show the prevalence of immunization status among children. The marginal likelihood effect was used to determine the impact of each category of the explanatory variable on one response category of immunization status. Ordinal logistic regression models were constructed, and the best-fitting model was selected to identify significant immunization status variables.

**Results:**

The immunization prevalence among children was 72.2% (34.2% fully immunized and 38.0% partially immunized), while about 27.8% of children were non-immunized. The fitted partial proportional odds model revealed that child immunization status was significantly associated with region afar (OR = 7.90; CI: 4.78–11.92), family planning use (OR = 0.69; CI: 0.54–0.88), residence (OR = 2.22;CI: 1.60–3.09), antenatal visit (OR = 0.73;CI: 0.53–0.99), and delivery place (OR = 0.65;CI: 0.50–0.84).

**Conclusions:**

Vaccinating children was a significant step forward in improving and protecting child health in Ethiopia, as the proportion of non-immunized children was about 27.8%. The study showed that the prevalence of non-immunization status among rural children was 33.6% and about 36.6% among children from non-educated mothers. As a result, it is agreeable that treatments are better to focus on targeting essential childhood vaccinations by promoting maternal education about family planning, antenatal visits, and maternal access to health care.

## Introduction

Immunization is a global success story and the most efficient and successful health intervention in health and development, saving millions of children’s lives each year. By bolstering the body’s natural defenses, vaccines lower the risk of contracting a disease [1–3]. Vaccines help individuals of all ages live longer, healthier lives by preventing more than 20 life-threatening diseases. According to global estimates, 1.5 million children die yearly from vaccine-preventable infections like measles due to inadequate vaccination coverage [4]. Vaccines prevent 2–4 million fatalities annually from diseases such as diphtheria, tetanus, pertussis, influenza, and measles. Immunization is an indisputable human right and essential to primary health care. Vaccines are also crucial for preventing and controlling infectious disease outbreaks, and they are critical in the fight against antimicrobial resistance and support global health security [5].

Vaccination has made significant benefits to public health, including the eradication of smallpox and the eradication of poliomyelitis in all but a few countries. Over the last few years, global vaccination coverage has been stable, with the percentage of children receiving recommended vaccines remaining constant. In 2019, over 85 percent of infants (116 million) received three doses of the diphtheria, tetanus, and pertussis (DTP3) vaccine, which protects them against infectious diseases that can cause serious illness, disability, or death. By 2019, 125 Member States have achieved at least 90% DTP3 immunization coverage [6]. The percentage of children receiving diphtheria, tetanus, and pertussis vaccine (DTP) is often used to indicate how well countries provide routine immunization services [7].

In less than a generation, the African Region has significantly improved vaccination coverage and reduced child mortality. Several illnesses, such as polio and maternal and neonatal tetanus, are on the verge of eradication and extinction, while the advent of new vaccines is ending age-old diseases [8]. In 2018, almost 870,000 Ethiopian children were denied life-saving measles, diphtheria, and tetanus immunization [9]. In Ethiopia, vaccination coverage increased from 42% in the 1990s to over 88% in 2013, and the number of diseases covered by the program increased from six to ten [10].

Several studies investigated the immunization status of children in Ethiopia using some set of variables, small-scale survey data, in specific areas or societies based on Ethiopian demographic and health survey (EDHS) 2011 and EDHS 2016 data without considering the ordinality properties of immunization status. They did not evaluate the impact of each explanatory variable on a single immunization status category [11–14]. As a result, the current study was carried out nationwide using the 2019 Ethiopian mini demographic and health survey (EMDHS) data to evaluate the immunization status of children 12–24 months and identify pertinent risk factors.

## Methods

### Study design and setting

The Ethiopian demographic and health survey (EDHS) is a comprehensive and nationally representative population and health survey conducted using a cross-sectional study design. The demographic health survey (DHS) collects a wide range of objectives and self-reported data with a strong focus on maternal and child health, nutrition, and mortality. Therefore the current study was based on the EMDHS data conducted in 2019.

### Data source

The data for this study was from the Ethiopian Mini Demographic and Health Survey (EMDHS), conducted from March to June 2019. The Ethiopian Public Health Institute (EPHI), in collaboration with the Central Statistical Agency (CSA) and the Federal Ministry of Health (FMoH), conducted the 2019 Ethiopian Mini Demographic and Health Survey, which is a nationally representative cross-sectional survey. The World Bank, the United States Agency for International Development (USAID), and the United Nations Children’s Fund provided funding for the 2019 EMDHS [15].

The sample frame for the 2019 EMDHS is a composite of all census enumeration areas (EAs) created for the 2019 Ethiopian Population and Housing Census (EPHC) by the Central Statistical Agency (CSA). The census frame lists all 149,093 EAs designed for the EPHC 2019. An EA is a geographical region that encompasses 131 households on average. In two stages, the 2019 EMDHS sample was stratified and selected. There were 21 sampling strata in each region, divided into urban and rural areas [16].

A total of 305 enumeration areas (EAs) were chosen in the first stage, with probability proportional to EA size. The second stage involved systematic sampling in selecting 30 households per EA. Immunization data was gathered from vaccine card records; if a vaccine card was unavailable, mothers were asked to recall their child’s vaccination history. If vaccination was not documented on the Infant Immunization Card or the health card, the mother was asked to remember if it had been given [16, 17].

In all selected households, the immunization status of 2,060 children aged 12–24 months was collected, of which 1,843 children were successfully reported [17].

### Data extraction method

The Ethiopian mini demographic and health survey data were taken from the demographic and health survey program website (http://www.dhsprogram.com) after gaining consent from the EDHS program. Data extraction, cleaning, variable selection, and recoding of some categorical variables were accomplished based on the existing literature using SPSS as the data were accessed by SPSS format.

### Inclusion-exclusion criteria

Children aged 12 to 24 months with known vaccination status (basic vaccines) were included in the study, while children whose vaccination status was unknown for some reason were excluded.

### Ethical approval and consent to participate

Ethical approval did not require because the study used a public survey dataset freely available online at http://www.dhsprogram.com. However, to access the data, we had to obtain permission and an approval letter from the DHS program via an online request.

### Variable measurements

#### Dependent variable

The outcome variable was the immunization status of children aged 12 to 24 months. It was calculated based on the vaccination status of each child. Fully vaccinated children received one dose of Bacillus Chalmette Guerin (BCG), three doses of diphtheria-tetanus-pertussis (DPT), three doses of the polio vaccine, and one dose of the measles vaccine [18, 19]. In contrast, partially vaccinated children received some but not all of the essential vaccines, and unvaccinated children received none.

#### Explanatory variables

Explanatory variables were selected based on theoretical considerations and research on factors affecting childhood immunization status. Previous studies used to form categories for naturally continuous and discrete variables [13, 19, 20]. Socioeconomic, demographic, and health-related factors are the independent variables used in the study. Those are age of mothers (15–24, 25–34, 35–49), region (Tigray, Amhara, Oromia, Somali, Benishangul, SNNPR, Gambela, Harari, Addis Ababa, and Dire Dawa), residence (urban and rural), parity (0–3, 4–6, > 6), marital status (unmarried, married), wealth index (poor, medium, rich), mothers education (no education, primary, secondary or above), religion (orthodox, Muslim, protestant or others), family size (less or equal to 5, more than 5), number of births in last five years (less or equal to one, more than one), delivery place (home/others and health institutions), ACV (no/don’t know and yes), birth order (first, second to fourth and less or equal to five), and family planning use (yes and no).

### Statistical analysis

Before starting the preliminary analysis, the extracted data were checked for completeness and consistency. The data were analyzed using STATA version 15 because we have some skills of using STATA and prefer it for ordinal data analysis. Data were weighted to account for differences in stratum selection and nonresponse probabilities. Both descriptive and inferential statistical methods were used to present the data. The immunization status of children aged 12 to 24 months was shown in descriptive statistics using frequency distributions and percentages.

The chi-square test examined the association between each explanatory factor and immunization status. Variables with a p-value less than 0.15 in the bivariate analysis were included in the final multivariable logistic regression analysis. The explanatory variables were examined for multicollinearity using the variance inflation test (VIF less than 10). Still, no co-linearity was found among the candidate variables (all candidates had a VIF value of less than 4). The factors of immunization status were identified using the ordinal logistic regression approach. Variables with p-values less than 0.05 were considered to have a statistically significant association with immunization status in the final model. The strength of the relationship was assessed using an odds ratio with a 95% confidence interval.

#### Ordinal logistic regression model

Logistic regression is a popular modeling approach for predicting the value of a categorical dependent variable with one or more independent variables [21]. Depending on the nature of the categories of the response variables, logistic regression models were divided into binary, multinomial, and ordinal models [22]. The ordinal logistic regression model is a type of logistic model used to examine ordinal dependent variables with more than two categories. The most commonly used ordinal logistic regression models are the continuation ratio, adjacent category, partial proportional, and proportional odds models [23]. The proportional odds model (POM) estimates the probability of being at or below a certain level of the response variable. It considers the likelihood of both this event and all previous events. Other unique ordinal models are used to find significant explanatory variables when the proportionality assumption, which states that the relationship between the independent variables and the dependent variable does not vary with the categories of the dependent variable, is not met [21].

The generalized ordered logit model (GOLM) is used when the proportional odds assumption is fully or partially relaxed for the explanatory variables. At the same time, the partial proportional odds model (PPOM) is used when the proportional odds assumption is satisfied for some but not all explanatory variables. The continuation ratio logistic model (CRM) contrasts the likelihood of responding to a particular category with the probability of responding to a higher response. When developing a logit for adjacent categories, the categorization of the response variable is taken into account, and logits are calculated for each pair of categories [24].

#### Parameter estimation

All ordinal models defined above were fitted to the data set using STATA (version 15). The POM was fitted using "ologit" stata command, and then the "Brant" test was used to evaluate the parallel line assumption. For ordinal logistic regression, the model parameters are estimated using maximum likelihood estimation (MLE) techniques. In general, the method of maximum likelihood produces values of the unknown parameters that best match the predicted and observed probability values.

#### Model selection

The log-likelihood values are used to compare the ordinal logistic model (the model with a higher log-likelihood, the better fit the model). Both Akaki and Baye’s are used to compare models. The model with the smallest absolute Akaki information criterion (AIC) and Bayesian information criterion (BIC) statistic is considered the best model. The Pearson and deviance goodness-of-fit test was used to measure the goodness of fit for the model [25].

#### Marginal effects

Marginal effects are a popular technique for making the effects of variables in nonlinear models more understandable. After the other factors in the model have been kept constant, the marginal effect (ME) for categorical variables shows how the probability of the response changes when the categorical variable changes from one category to another [26].

In addition, mean values are only one of many alternative value sets that can be used and selecting a value set that only some individuals can have seems problematic. Many researchers prefer marginal probability effects (MPE) for these and other reasons. Some argue that it is better to use the actual observed values for the variables whose values are not otherwise fixed when calculating the predicted values rather than the mean values. Using the fixed and observed values of the variables, we then calculate a predicted probability for each case and, finally, the average of the expected values. MPE is a typical manner of responding to the question, "What effect does the predictor have on the likelihood of the event occurring? [27].

## Results

### Socio-demographic and other characteristics of children

This study is based on data from the 2019 EMDHS. A total of 1,843 children aged 12 to 24 months were enrolled in the study; of these, 630 (34.2%) had received all recommended vaccines, 1330 (72.2%) had received some or all recommended vaccines, and 513 (27.8%) had received none of the recommended vaccines (Table 1).

**Table 1:** Proportions of immunization status among children aged between 12–24 months, EMDHS 2019 (*n = 1*,*843*).

Immunization status	Freq.	percent	Com. Percent
Fully immunized	630	34.2	34.2
Partially immunized	700	38.0	72.2
Not immunized	513	27.8	100

Table 2 shows the distribution of immunization status by demographic, socioeconomic, and other characteristics among children aged 12 to 24 months. Of the total 1,843 children in the sample, 1,376 (74.7%) were from rural areas, and the remaining children were from urban areas. Among children aged 12–24 months, 28.6%, 37.8%, and 33.6% were fully immunized, partially immunized, and not immunized, respectively. In rural areas, about 33.6% of children were not immunized, while about 10.7% were not vaccinated in urban areas. The percentage of fully and partially immunized was higher among children from mothers aged between 35 and 49 years, but the rate of none immunized was higher among children from mothers aged 15–24.

**Table 2:** Socio-demographic and other characteristics of children’s immunization status, mini EDHS 2019 (*n = 1*,*843*).

Variables	Full immunized Count (%)	Partially immunized Count (%)	Not immunized Count (%)	Total
**Age of mothers**				
15–24	153(27.5)	239(42.9)	165(29.6)	557
25–34	360(37.2)	348(35.9)	259(26.8)	967
35–49	117(36.7)	113(35.4)	89(27.9)	319
**Residence**				
Urban	237(50.8)	180(38.5)	50(10.7)	467
Rural	393(28.6)	520(37.8)	463(33.6)	1376
**Educ. Level**				
No education	257(28.2)	322(35.2)	334(36.6)	913
Primary	237(36.5)	261(40.1)	152(23.4)	650
Sec/above	136(48.6)	117(41.8)	27(9.6)	280
**Wealth index**				
Poor	227(25.6)	300(33.9)	358(40.5)	885
Middle	88(32.5)	114(42.0)	69(25.5)	271
Rich	315(45.9)	286(41.6)	86(12.5)	687
**Antenatal visit**				
No /don’t know	71(14.9)	147(30.8)	259(54.3)	477
Yes	559(40.9)	553(40.5)	254(18.6)	1366
**Religion**				
Orthodox	287(50.2)	201(35.1)	84(14.7)	572
Muslim	243(27.3)	334(37.5)	314(35.2)	891
Others	100(26.3)	165(43.4)	115(30.3)	380
**Parity**				
0–3	391(37.3)	407(38.8)	251(23.9)	1049
4–6	177(31.4)	205(36.4)	181(32.2)	563
> = 6	62(26.8)	88(38.1)	81(35.1)	231
**Births in 5 years**				
**≤**1	406(42.5)	367(38.4)	183(19.1)	956
>1	224(25.3)	333(37.5)	330(37.2)	887
**Family planning**				
No	311(26.4)	343(36.9)	432(36.7)	1177
Yes	319(47.9)	266(39.9)	81(12.2)	666
**Birth order**				
First	155(37.6)	181(43.9)	76(18.5)	412
Two to four	313(36.3)	308(35.7)	241(28.0)	862
Five and above	162(28.5)	211(37.1)	196(34.4)	569
**Family size**				
**≤ 5**	360(40.0)	340(37.8)	200(22.2)	900
**> 5**	270(28.6)	360(38.2)	313(33.2)	943
**Marital status**				
Unmarried	43(34.4)	49(39.2)	33(26.4)	125
Married	587(34.2)	651(37.9)	480(27.9)	1718
**Delivery place**				
Home/others	180(21.5)	282(33.7)	374(44.7)	836
Health institution	450(44.7)	418(41.5)	139(13.8)	1007
**Region**				
Tigray	95(59.0)	56(34.8)	10(6.2)	161
Afar	19(9.5)	70(34.8)	112(55.7)	201
Amhara	88(49.7)	51(28.8)	38(21.5)	177
Oromia	61(26.6)	104(45.4)	64(28.0)	229
Somali	20(12.2)	53(32.3)	91(55.5)	164
Benishangul	68(40.7)	62(37.1)	37(22.2)	167
SNNPR	51(25.5)	81(40.5)	68(34.0)	200
Gambela	47(30.5)	66(42.9)	41(26.6)	154
Harari	48(34.2)	53(37.9)	39(27.9)	140
Addis Ababa	73(64.0)	37(32.5)	4(3.5)	114
Dire Dawa	60(44.1)	67(49.3)	9(6.6)	136

Children of mothers who participated in antenatal care (ANC) had a higher percentage of fully and partially immunized. In comparison, children of mothers who did not participate in the ANC had a higher percentage of non-immunized (Table 2).

As mothers’ income levels increased, the percentage of children with full and partial immunization status increased; conversely, as mothers’ income levels decreased, the percentage of none immunized children decreased. Women who gave birth in a health facility had increased rates of full and partial immunization of their children.

### Ordinal logistic regression analysis

Before fitting the ordinal logistic regression model, a chi-square test for association was performed, and then significant explanatory factors were added to the model at a 15% significance level. The proportional odds model was discarded due to violation of the parallelism assumption by the Brant test (chi-square = 82.47, p-value = 0.001), and the data were then fitted with partial proportional, generalized ordered logit, continuation ratio, and adjacent category logit models. Finally, a model comparison was performed based on information criterion and log-likelihood values. Due to the lowest information criterion values, the PPOM was considered the best-fitting model (Table 3).

**Table 3:** AIC and BIC values for all ordinal models.

Model	POM	PPOM	GOM	CRM	ACM
**Observations**	1843	1843	1843	1843	1843
**Df**	31	41	60	60	60
**Akaki’s IC**	3552.19	3480.441	3500.22	3503.05	3505.09
**Bayesian IC**	3723.28	3706.73	3831.37	3834.19	3836.24

The Pearson test for goodness of fit confirmed that the PPOM was the best fit for the data (chi-square = 3032.2, p-value = 0.546). Thus, PPOM was used to identify significant determinants of childhood immunization status, and parameter estimates were interpreted for the significant predictors at a 5% significance level.

#### Results of Partial Proportional Odds Model (PPOM)

Table 4 shows two contrasting result panels. The contrasts are fully immunized versus partially and non-immunized and fully and partially immunized versus non-immunized.

**Table 4:** Revealed the results of the parameter estimates under PPOM, EMDHS 2019 (*n = 1*,*843*).

Predictors		Fully immunized				Partially immunized			
		Coef.	p-value	OR	95%CI	Coef.	P-value	OR	95%CI
Region(Ref: Tigray)	Afar	2.070	0.001	7.90	4.78–11.92	2.070	0.001	7.90	4.79–11.90
	Amhara	-0.633	0.005	0.53	0.45–0.61	-1.120	0.001	0.33	0.29–0.37
	Oromia	1.590	0.001	4.88	3.06–7.79	1.590	0.001	4.88	3.06–7.79
	Somali	1.610	0.001	5.01	3.06–8.55	1.610	0.001	5.01	3.06–8.55
	Benishangul G	1.330	0.003	3.77	1.57–8.98	1.330	0.003	3.76	1.58–8.98
	SNNP	1.220	0.001	3.38	1.35–7.34	1.257	0.001	3.52	2.67–6.09
	Gambela	1.550	0.001	4.69	2.02–10.88	1.550	0.001	4.69	2.02–10.88
	Harari	1.410	0.035	4.09	1.14–6.02	1.410	0.035	4.09	1.14–6.02
	Addis Ababa	-0.580	0.001	0.56	0.34–0.78	-0.580	0.001	0.56	0.34–0.78
	Dire Dawa	1.130	0.037	3.08	1.07–8.89	1.130	0.037	3.08	1.07–8.89
Educational Level (Ref: no education)	Primary	-0.072	0.532	0.93	0.74–1.16	-0.072	0.532	0.93	0.75–1.17
	Sec/higher	-0.340	0.002	0.71	0.49–0.93	-0.340	0.002	0.71	0.49–0.93
Family planning (ref: no)	Yes	-0.368	0.003	0.69	0.54–0.88	-0.855	0.001	0.43	0.32–0.56
Residence (ref: urban)	Rural	0.801	0.001	2.22	1.60–3.09	0.196	0.017	1.22	1.03–1.78
Family size (Ref: < = 5)	>5	0.110	0.393	1.11	0.86–1.43	0.110	0.393	1.12	0.87–1.43
Parity (Ref: 0–3)	4–6	0.133	0.441	1.14	0.81–1.60	0.133	0.441	1.14	0.82–1.61
	> = 6	0.432	0.041	1.54	1.24–2.51	0.432	0.041	1.54	1.24–2.51
Age of mothers (Ref:15–24)	25–34	-0.799	0.001	0.45	0.35–0.59	-0.799	0.001	0.45	0.35–0.59
	35–49	-0.873	0.001	0.42	0.29–0.61	-0.873	0.001	0.42	0.29–0.61
Births in last 5 years Ref: < = 1	>1	0.388	0.003	1.48	1.14–1.90	0.189	0.021	1.21	1.05–1.46
Birth order (Ref: first)	2 to 4	-0.282	0.096	0.75	0.54–1.05	0.166	0.358	1.18	0.83–1.68
	> = 5	0.088	0.710	1.09	0.69–1.73	0.087	0.710	1.09	0.69–1.73
Wealth Index (Ref: poor)	Middle	-0.031	0.831	0.97	0.76–1.25	-0.031	0.831	0.97	0.76–1.25
	Rich	-0.472	0.003	0.63	0.18–0.91	-0.472	0.003	0.63	0.18–0.91
Delivery Place (Ref: Home/other)	Health institution	-0.434	0.001	0.65	0.50–0.84	-0.781	0.001	0.46	0.35–0.60
Antenatal Visits (Ref: no)	Yes	-0.319	0.043	0.73	0.53–0.99	-0.648	0.001	0.52	0.39–0.68
Marital status (Ref: unmarried)	Married	0.291	0.152	1.34	0.89–1.99	0.291	0.153	1.34	0.91–1.99
Religion (Ref: Orthodox)	Muslim	-0.301	0.055	0.74	0.54–1.01	-0.301	0.055	0.74	0.54–1.01
	Protestant/Other	0.169	0.339	1.18	0.84–1.68	-0.248	0.158	0.78	0.55–1.10
	Constant	-0.511				-1.419			

Key: LR chi2 (39) = 548.39 Prob >chi2 = 0.001

The variable’s region (Amhara), residency, family planning use, number of births in the last five years, delivery place, and antenatal care visit violated the parallel lines’ assumption in the partial proportional odds model. The model, therefore, allows the coefficients of these variables to vary across the response categories. From the PPOM results, region, mother’s education, family planning use, residency, parity, age of mothers, number of birth in the last five years, wealth index, delivery place, and antenatal care visits were significantly related to the immunization status of children aged between 12–24 months.

#### Predictors that violate the parallel line assumption

The partial proportional odds model result showed that, when all other variables were held constant, a child in Amhara was roughly 47% (OR = 0.53, p-value = 0.005) less likely to be partially or not immunized than a child in Tigray. The fitted model revealed that children in rural locations were 2.2 (OR = 2.22, p-value = 0.001) and 1.2 (OR = 1.22, p-value = 0.017) times more likely to report partially or not immunized and not immunized, respectively, as compared with urban children.

This study’s result revealed that compared to children whose mothers didn’t use family planning, children whose mothers used family planning were about 31% (OR = 0.69, p-value = 0.003) less likely to be partially or none immunized rather than fully immunized. Similarly, compared to a child whose mother didn’t use family planning, a child whose mother used family planning was approximately about 57% (OR = 0.43, p-value = 0.001) less likely to be none immunized. The fitted model also showed that, as compared to a child whose mother didn’t visit antenatal care, a child whose mother visited antenatal care was about 27% (OR = 0.73, p-value = 0.043) and about 48% (OR = 0.521, p-value = 0.001) times less likely to report partially or non-immunized and non-immunized respectively. A mother who delivered in a health institution was about 35% (OR = 0.65, p-value = 0.001), and about 54% (OR = 0.46, p-value = 0.001) decreased their child to be partially or non-immunized and non-immunized, respectively. Holding all other variables constant, compared with children whose mothers had one or no births in the last five years, children whose mothers had more than one birth in the last five years were 1.5 (OR = 1.48, p-value = 0.003) and 1.2 (OR = 1.21, p-value = 0.021) times more likely to report partially or non-immunized and non-immunized respectively.

#### Predictors that do not violate the parallel line assumption

When all other variables keep constant, a child from Afar was more likely to report a higher risk of being non-immunized than a child in Tigray (OR = 7.90, p-value = 0.001). According to the results of PPOM, a child in Somali (OR = 5.01, p-value = 0.001), Oromia (OR = 4.88, p-value = 0.001), Gambela (OR = 4.69, p-value = 0.001), and Dire Dawa (OR = 3.08, p-value = 0.037) was also more likely to report worse immunization status than a child in Tigray region. Compared to children in Tigray, children in Addis Ababa were roughly 44% (OR = 0.56, p-value 0.001) less likely to report partial/non- immunization status.

The fitted model showed that compared with children from low-income families, children from rich families were about 37% (OR = 0.63, p-value = 0.003) less likely to report worse immunization status. Compared to children from non-educated mothers, the fitted model showed that children from sec/higher educated mothers were about 1.4 (OR = 0.71, p-value = 0.002) times more likely to report better immunization status. Keeping all other variables constant, children from mothers aged 25–34 and 35–49 were 2.2 (OR = 0.45, p-value = 0.001) and 2.4 (OR = 0.42, p-value = 0.001) times more likely to be partially or fully immunized than children from mothers aged 15–24 years respectively. Similarly, children from mothers with parity more/equal to 6 were 1.5 (OR = 1.54, p-value = 0.041) times more likely to be partial or non-immunized than children with parity less/equal to three.

### Marginal effects

The marginal probability effects result (Table 5) revealed significant marginal effects for the region (Afar, Oromia, Somali, Addis Ababa, and Gambela), educational level (sec/higher), family planning, residence, parity (> = 6), wealth index (rich), antenatal visit, delivery place, and the number of births in last five years (more than one).

**Table 5:** Average marginal probability effects (MPE) of predictors on anemia levels, EMDHS 2019 (*n = 1*,*843*).

Predictors		Fully immunized		Partially immunized		Non- immunized	
		MPE1	P-value	MPE2	P-value	MPE3	P-value
Region	Afar	-0.503	0.001	-0.151	0.017	0.352	0.001
	Amhara	0.155	0.003	0.056	0.212	-0.099	0.001
	Oromia	-0.371	0.001	-0.205	0.001	0.166	0.001
	Somali	-0.443	0.001	0.196	0.001	0.247	0.001
	Benishangul	-0.319	0.001	0.194	0.001	0.125	0.034
	SNNPE	-0.396	0.001	0.094	0.063	0.303	0.001
	Gambela	-0.363	0.001	0.204	0.001	0.159	0.011
	Harari	-0.428	0.004	0.201	0.001	0.228	0.203
	AddisAbaba	0.285	0.001	0.182	0.001	-0.104	0.015
	Dire Dawa	-0.275	0.027	0.178	0.005	0.097	0.133
Education Level	Primary	0.016	0.533	-0.003	0.541	-0.013	0.532
	Sec/higher	0.175	0.001	-0.022	0.161	-0.053	0.047
Family planning	Yes	0.079	0.003	0.058	0.030	-0.138	0.001
Residence	Rural	-0.182	0.000	-0.151	0.001	0.031	0.300
Parity (TNCEB)	4–6	-0.028	0.440	0.007	0.440	0.022	0.442
	> = 6	-0.088	0.030	0.011	0.043	0.077	0.009
Age of mothers	25–34	0.157	0.001	-0.011	0.197	-0.147	0.001
	35–49	0.175	0.001	-0.017	0.169	-0.157	0.001
Wealth Index	Middle	0.006	0.831	-0.002	0.833	-0.005	0.831
	Rich	0.098	0.002	0.141	0.001	-0.043	0.013
Antenatal Visit	Yes	0.066	0.035	0.052	0.09	-0.118	0.001
Delivery place	Health institution	0.092	0.001	0.039	0.171	-0.131	0.001
Birth in last 5 years	>1	-0.083	0.003	0.064	0.014	0.078	0.021

The fitted MPE depicted that as a region shifts from Tigray to Somali and Afar, the likelihood of children in Somali and Afar being fully immunized decreases by 44 (MPE = -0.443, p-value = 0.001) and 50 (MPE = -0.503, p-value = 0.001) percentage points, respectively. Comparing children in Oromia to children in Tigray, the probability of children in Oromia being fully immunized drops by about 37 (MPE = -0.371, p-value = 0.001) percentage points. Children’s chances of being fully immunized increased by 15 (MPE = 0.155, p-value = 0.003) and 28 (MPE = 0.285, p-value = 0.001) percentage points as we went from Tigray to Amhara and Addis Ababa, respectively.

The result of MPE showed that the probability of children from sec/higher educated mothers being fully immunized would increase by approximately 18 (MPE = 0.175, p-value = 0.001) percentage points compared to children from uneducated mothers. Compared to children whose mothers do not use family planning, the probability that children whose mothers use family planning are fully immunized raised by about 8 (MPE = 0.079, p-value = 0.003) percentage points, whereas the probability of being non-immunized drops by about 14 (MPE = -0.138, p-value = 0.001) percentage points.

Based on the fitted MPE model, as residence changes from urban to rural, children’s probability of being fully immunized would fall by approximately 18 (MPE = -0.182, p-value = 0.001) percentage points. As compared to children from mothers with parity less or equal to 3, children’s probability who have six or more children being fully immunized would decrease by approximately 9 (MPE = -0.088, p-value = 0.030) percentage points, whereas the probability of being non-immunized would increase by approximately 8 (MPE = 0.077, p-value = 0.009) percentage points.

Holding all other variables constant, the likelihood of children from mothers aged 25–34 and 35–49 being fully immunized would increase by 16 (MPE = 0.157, p-value– 0.001) and 18 (AME = 0.175, p-value = 0.001) percentage points, respectively, compared to children from mothers aged 15–24 years. Children from rich families have a higher chance of fully immunizing by about 10 (MPE = 0.098, p-value = 0.002) percentage points than children from low-income families. As compared with children from antenatal visitor mothers, children from non-visitor mothers’ chance of being fully immunized increased by about 7 (MPE = 0.066, p-value = 0.035) percentage points, while the likelihood of being non-immunized falls by about 12 (AME = -0.118, p-value = 0.001) percentage points. The probability of being fully immunized among children from mothers whose delivery place at a health institution was increased by about 9 (MPE = 0.092, p-value = 0.001) percentage points, whereas the chance of being non-immunized decreased by 13 (MPE = -0.131, p-value = 0.001) percentage points.

## Discussion

In this study, the immunization status of children aged 12 to 24 months were assessed and ranked as an ordinal response depending on the number of vaccines the child received. Overall, 72.2% of children were immunized, with 34.2% receiving all recommended vaccines and 38% receiving only some. Only 27.8% of infants had received none of the recommended vaccinations. Model comparisons were performed after fitting the data with proportional, partial proportional odds, generalized ordered logit, adjacent category logit, and continuation ratio models. As a result, the PPOM model used to find significant factors for children’s immunization status is the best fit based on IC values. Parameter estimates were presented and explained for the relevant predictors (at a 5% significance level). The region where children live, place of residence, maternal age, education level, wealth index, number of births in the past five years, family planning use, parity, place of delivery, and prenatal visitation are all important variables associated with children’s immunization status.

The study discovered that children’s immunization status was greatly influenced by their region. This finding is in line with research from Ethiopia [13, 19, 28], which found that non-immunization was higher in the Afar, Oromia, and Somali regions. Compared to children in urban areas, children from rural areas were more likely to be in the worst category of immunization status. This finding is consistent with research conducted in Ethiopia [28, 29] and Bangladesh [14]. The reason could be a lack of health facilities, poor health-seeking behavior, or a poor immunization program.

The study also discovered maternal education is a potential indicator of child immunization status. Children of mothers with secondary or higher education were less likely to have poorer immunization status than children of uneducated mothers. Studies from Ethiopia [30] and Indonesia [31] confirm this finding. This could be because educated mothers know more about protecting their children’s health and have a better understanding of child immunization. It is better to support and promote women’s education. The results of the Ethiopian privilege research showed that children from affluent families were at the lowest risk of being vaccinated compared with children from impoverished households. Studies in developing nations such as Ethiopia [19], Nigeria [32], and Bangladesh [33] back this conclusion. The result could be because having a low salary means having less probability of getting health facilities and health workers.

This study’s findings suggest that not only ANC visiting but also the mother’s delivery place can help with children’s immunization status. This research is in line with previous research in Ethiopia [34] and Nigeria [35, 36]. Women who attended ANC follow-up were encouraged to protect their children by health professionals, and prenatal care counseling can assist women in remembering to immunize their children. Another study finding indicated that using family planning increased the risk of children being immunized. This result is consistent with past studies [37, 38]. The likely explanation for this link is that women require more information about keeping children healthy.

The findings of this study showed that the higher the total number of children ever born and the total number of births in the last five years, the less likelihood of children being immunized. This result agreed with studies in Ethiopia [12, 30] and Ghana [39]. This could be because having more children could lead to food instability and poverty in the home, and women have more information about the use of immunization. This study also indicated that the mother’s delivery place was a significant factor in child immunization. Mothers with the delivery place of health institutions increased the likelihood of child immunization. This conclusion is in line with the studies in Ethiopia [13, 29] and Nigeria [36]. It may be because the mothers get enough information and basic advice about child health from health professionals.

The study also examined the marginal effect of each explanatory variable on single immunization status. The result was that the categories of Somali, Oromia, Afar, higher education, mother’s age between 35 and 49 years, rural residence, and the total number of children born over six had a high impact on the child’s single immunization response.

### Strength and limitations

The strength could be the high response rate, and the study was based on numerous variables considering the ordinal property of immunization status. The study also shows the marginal effects of each explanatory variable. Due to numerous missing values, many important explanatory variables, such as the HIV status of women, were not included in this study. Since the EDHS is a questionnaire-based survey that relies on respondents’ recollections, recall bias in the data could be a weakness in this study.

## Conclusions

Non-immunization was present in 27.8% of children aged between 12–24 months, according to the findings. According to the results of the fitted partial proportional odds model, children’s region, residence, antenatal care visits, parity, and age of mothers, the number of births in the last five years, maternal education, wealth index, and delivery place were all found to be significantly associated with child immunization status. Antenatal care visits and education should aim to raise awareness and the importance of child health protection by promoting family planning.

Policy interventions aimed at increasing the immunization rate among children could be implemented to improve access to health care by providing basic services concerned with child health. Further research should look at multilevel analysis to deal with the hierarchical nature of the data and reduce regional discrepancies in the immunization status among children in Ethiopia.

## Abbreviations

POM: proportional odds model
PPOM: partial proportional odds model
GOLM: generalized ordered logit model
CRM: continuation ratio model
LRT: likelihood ratio test
MLE: maximum likelihood estimate
VIF: variance inflation factor
ME: Marginal Effect
MPE: marginal probability effect
EDHS: Ethiopian demographic health survey
EMDHS: Ethiopian mini demographic health survey
EPHC: Ethiopian population and housing census
EA: enumeration area
SNNPR: Southern nations nationality and people region
WHO: world health organization
USAID: United States agency for international development
FMoH: federal ministry of health
EPHI: Ethiopian public health institute
ANC: antenatal care
BCG: Bacillus Chalmette Guerin
